# Efficient anchoring of alien chromosome segments introgressed into bread wheat by new *Leymus racemosus* genome-based markers

**DOI:** 10.1186/s12863-018-0603-1

**Published:** 2018-03-27

**Authors:** Offiong Ukpong Edet, June-Sik Kim, Masanori Okamoto, Kousuke Hanada, Tomoyuki Takeda, Masahiro Kishii, Yasir Serag Alnor Gorafi, Hisashi Tsujimoto

**Affiliations:** 1 Arid Land Research Center, Tottori University, Tottori, Japan; 2 RIKEN Center for Sustainable Resource Science, Tsukuba, Ibaraki, 305-0074 Japan; 3 Center for Bioscience Research and Education, Utsunomiya University, Utsunomiya, Japan; 4 Department of Bioscience and Bioinformatics, Kyushu Institute of Technology, Kitakyushu, Japan; 5 International Maize and Wheat Improvement Center (CIMMYT), El Batan, Mexico; 6 Agricultural Research Corporation (ARC), Wad Madani, Sudan; 7 United Graduate School of Agricultural Sciences, Tottori University, Tottori, Japan

**Keywords:** *Leymus racemosus* (1), Molecular markers (2), DArT-seq (3), Genome sequencing (4), Chromosome introgression lines (5), Salinity stress (6), Triticeae (7)

## Abstract

**Background:**

The tertiary gene pool of bread wheat, to which *Leymus racemosus* belongs, has remained underutilized due to the current limited genomic resources of the species that constitute it. Continuous enrichment of public databases with useful information regarding these species is, therefore, needed to provide insights on their genome structures and aid successful utilization of their genes to develop improved wheat cultivars for effective management of environmental stresses.

**Results:**

We generated de novo DNA and mRNA sequence information of *L. racemosus* and developed 110 polymorphic PCR-based markers from the data, and to complement the PCR markers, DArT-seq genotyping was applied to develop additional 9990 SNP markers. Approximately 52% of all the markers enabled us to clearly genotype 22 wheat-*L. racemosus* chromosome introgression lines, and *L. racemosus* chromosome-specific markers were highly efficient in detailed characterization of the translocation and recombination lines analyzed. A further analysis revealed remarkable transferability of the PCR markers to three other important Triticeae perennial species: *L. mollis, Psathyrostachys huashanica* and *Elymus ciliaris*, indicating their suitability for characterizing wheat-alien chromosome introgressions carrying chromosomes of these genomes.

**Conclusion:**

The efficiency of the markers in characterizing wheat-*L. racemosus* chromosome introgression lines proves their reliability, and their high transferability further broadens their scope of application. This is the first report on sequencing and development of markers from *L. racemosus* genome and the application of DArT-seq to develop markers from a perennial wild relative of wheat, marking a paradigm shift from the seeming concentration of the technology on cultivated species. Integration of these markers with appropriate cytogenetic methods would accelerate development and characterization of wheat-alien chromosome introgression lines.

**Electronic supplementary material:**

The online version of this article (10.1186/s12863-018-0603-1) contains supplementary material, which is available to authorized users.

## Background

*Leymus racemosus* (mammoth wild rye) and other wild relatives of wheat in the tribe Triticeae (family Poaceae) have, over time, been variously utilized for breeding of hexaploid wheat [1–11]. A typical member of the tribe Triticeeae, *L. racemosus* is a tetraploid species with 14 linkage groups and seven basic chromosomes in each of its genomes (2n = 4× = 28; NsNsXmXm) [12].The genus *Psathyrostachys* is accepted to be the progenitor source of the Ns genome, but the progenitor of Xm genome has not been ascertained although some recent reports claim that tetraploid *Leymus* species are segmental polyploids having variant Ns genomes (Ns_1_Ns_2_) of *Psathyrostachys* [13, 14]. *Leymus racemosus* is a renowned sturdy species with high potentials for breeding of bread wheat [15–17].

Mining of useful genes from wild genetic resources, especially the tertiary gene pool, through distant hybridization, to broaden the genetic base of elite cultivars of bread wheat is expected to continue, considering the current trend in global climatic change, accompanied by new strains of pests and disease pathogens. This strategy is, however, largely hindered by linkage drag and low rate of success in distant hybridization, which, in this age of next-generation sequencing (NGS) technologies and improved interspecific hybridization techniques, can effectively be managed. While in vitro culture techniques, example embryo rescue, and induction of homoeologous chromosome recombination have been employed to achieve successful distant hybridization and useful gene recombination, integration of appropriate molecular markers into breeding programs to conduct marker-assisted backcrossing can immensely assist in selecting against deleterious genes, hence fast-tracking the process. Unfortunately, unlike their cultivated counterparts, whose genomes have been extensively analyzed**,** DNA sequence information and molecular markers of these wild species are limited or completely absent in some cases, culminating in a poor understanding of their genome structures and delay in cultivar development and adequate characterization. This dearth of information, which our research sought to address, largely accounts for the current underutilization of the rich diversity readily available for wheat breeding.

Plant breeders, in various attempts to deal with the aforesaid situation, have had to resort to applying available expressed sequence tags (ESTs) from a few perennial grasses and heterologous markers from annual cereals, example barley, to aid their work [18, 19], but the outcomes, although informative, are hardly satisfactory as a consequence of increased species divergence arising from mutations and other genetic events during speciation. To effectively harness useful genes from these all-important genetic resources, their genomic information base should be continually enriched to at least include data on outstanding species that can serve as representatives for their evolutionary close relatives. Efforts to achieve this much needed expansion have generated enormous molecular cytogenetic data, EST-SSR markers, EST linkage maps and other useful information [12, 19–23]. However, molecular markers developed from whole genome sequence information of these species are still lacking, making it difficult to adequately anchor alien chromosome segments in wheat-alien chromosome introgression lines (CILs). Also, the application of DArT-seq genotyping to study diversity and develop molecular markers from wild Triticeae species is yet to be accorded the popularity it deserves.

In this research, therefore, we applied PCR and DArT-seq to develop 8632 [110 PCR-based and 8522 DArT-seq (SNPs)] polymorphic markers from the genome of *Leymus racemosus.* We also developed additional 1468 CILs-based SNPs which are obvious polymorphisms resulting from the interaction between the alien chromosomes and the background/carrier. Our efforts extended to the application of 5196 (~ 52%) of all the markers to genotype 22 wheat-*L. racemosus* CILs and the analysis of the transferability of PCR-based markers to other Triticeae species, with emphasis on donor species whose genomes have not been sequenced. This is the first research reporting on the development of molecular markers from *L. racemosus* whole genome and RNA-seq, and the application of DArT-seq platform of wheat to develop DNA markers from its perennial wild relative.

## Results

### Development of *L. racemosus* polymorphic markers

**Fig. 1:**
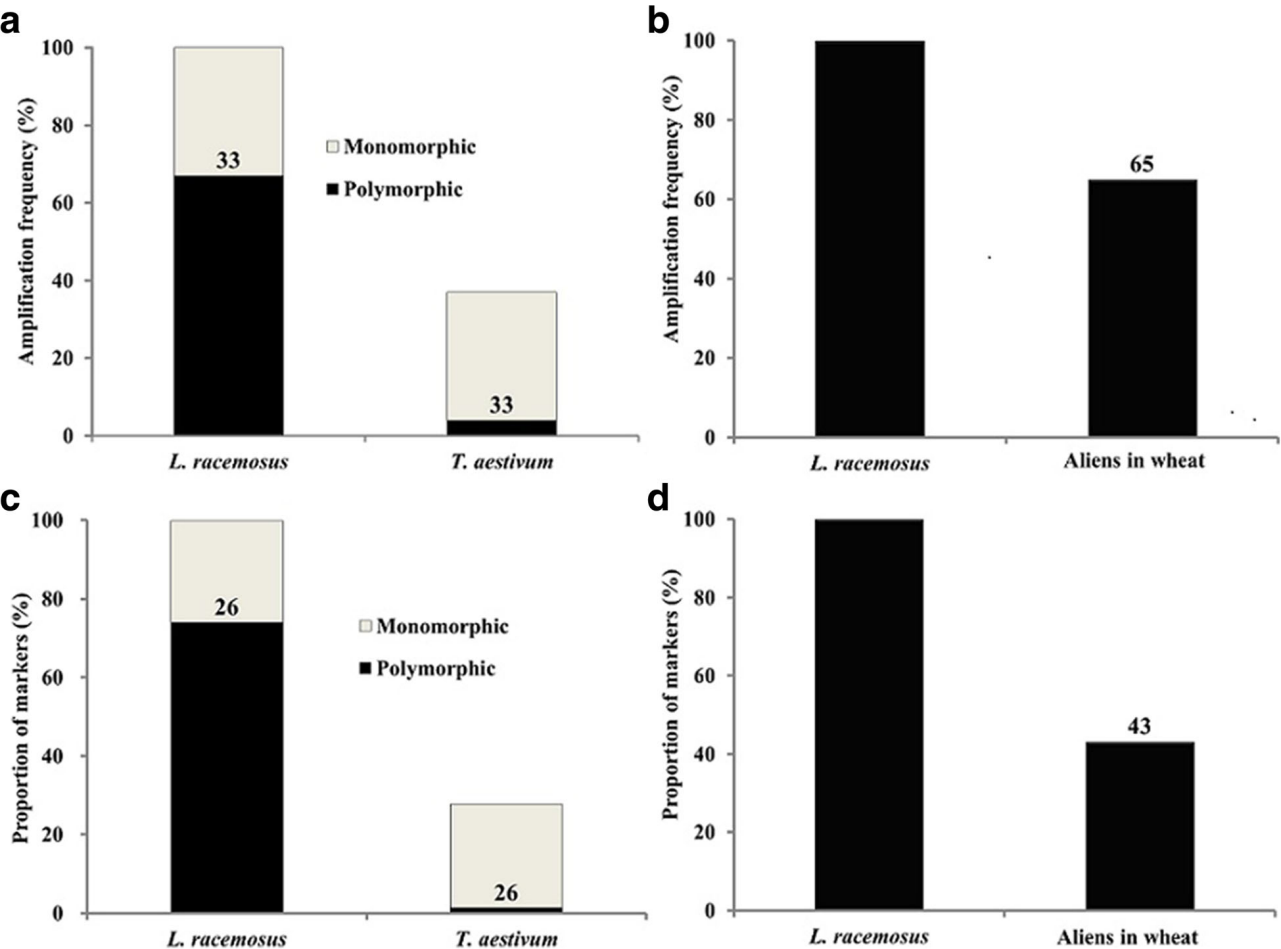
Analysis of markers located on *L. racemosus*, bread wheat and nine wheat-*L. racemosus* chromosome addition lines. **a** Amplification of 164 pre-screened *L. racemosus* PCR-based markers in *L. racemosus* and bread wheat genomes. **b** Amplification of 110 polymorphic *L. racemosus* PCR-based markers in nine wheat-*L. racemosus* addition lines, with *L. racemosus* genome as a positive control. **c** Differentiation of *L. racemosus* and bread wheat genomes using 11,570 SNPs. **d** Total SNPs **(**8522) located in nine wheat-*L. racemosus* addition lines

From a total of 294 primer sets screened by PCR, 164 sets (~ 56%) amplified *L. racemosus* genome. Out of the amplified markers, 110 (~ 67%) were polymorphic in wheat – absence or difference in size of bands in wheat – (Fig. 1a; Table 1). Six of the polymorphic markers showed size polymorphism, while 104 markers constituted presence/absence polymorphism. Also, out of 11,570 DArT-seq SNP markers filtered based on high call and reproducibility rates, 8522 (~ 74%) were polymorphic in wheat (absence of SNP alleles in wheat) –8430 SNPs were absent in our wheat cultivar, CS, while 92 were present but showed presence of both reference and SNP alleles in *L. racemosus* (Fig. 1c; Table 1). These 92 markers form part of the polymorphisms we observed between our CS and the reference CS genome sequence on DArT platform. Taken together, we developed a total of 8632 polymorphic markers from *L. racemosus* genome.

**Table 1:** Identification of wheat-*L. racemosus* chromosome introgression lines using polymorphic markers from *L. racemosus* genome

Genotype ID	Description	Chromosome constitution (2n)	Total number of markers		Chromosome-specific markers		
			PCR	DArT-seq	PCR	DArTseq	Total
TACBOW 0001	Disomic addition	21″ + 1″[Lr#A]	14	434	6	381	387
TACBOW 0003	Disomic addition	21″ + 1″[Lr#E]	4	46	1	1	2
TACBOW 0004	Disomic addition	21″ + 1″[Lr#F]	10	499	4	344	348
TACBOW 0005	Disomic addition	21″ + 1″[Lr#H]	20	528	12	410	422
TACBOW 0006	Disomic addition	21″ + 1″[Lr#I]	12	558	4	491	495
TACBOW 0007	Disomic addition	21″ + 1″[Lr#J]	17	511	9	450	459
TACBOW 0008	Disomic addition	21″ + 1″[Lr#K]	12	519	7	465	472
TACBOW 0009	Disomic addition	21″ + 1″[Lr#L]	18	647	8	525	533
TACBOW 0010	Disomic addition	21″ + 1″[Lr#N]	16	549	5	428	433
All aliens	–	–	**72**	**3656**	**56**	**3495**	**3551**
KT020–003 (CS)	*Triticum aestivum*	42	6	92	–	–	–
TACBOW 0112	*Leymus racemosus*	28	110	8522	–	–	–

*TACBOW* Tottori Alien Chromosome Bank of Wheat (Japan), *Lr Leymus racemosus*, *A, E, F, H, I, J, K, L, N* Arbitrary numbering of *L. racemosus* chromosomes;″: bivalent; Bold numbers: all markers located on alien chromosomes in wheat background in each category; CS: Chinese Spring

### Characterization of wheat-*L .racemosus* chromosome introgression lines with *L. racemosus* markers

About 65% (72 markers) of the polymorphic PCR markers amplified *L. racemosus* chromosomes in nine wheat-*L. racemosus* chromosome addition lines, while approximately 43% (3656 SNPs) of the DArT-seq (SNP) markers identified the nine alien chromosomes (Table 1; Fig. 1b and d). It should be noted here that we used only SNP markers from the DArT-seq data in our analysis, as silico DArT was less informative in analyzing the required polymorphism. This is because silico DArT data is binary (dominant), making it impossible to identify polymorphism as codominance (in our case, presence of both reference and SNP alleles), which we mostly utilized to genotype the chromosome introgression lines, since they have genome representations of wheat (alien chromosome recipient) and *L. racemosus* (alien chromosome donor).

### Development of *L. racemosus* chromosome-specific markers

We developed a total of 3551 chromosome-specific markers for the nine *L. racemosus* chromosomes in wheat genetic background, and the number of the specific markers per chromosome ranged between two in Lr#E and 533 in Lr#L (Table 1; Additional files 1, 2, 3, 4, 5, 6, 7, 8 and 9). The large number of markers on each chromosome enabled us to reliably differentiate the nine wheat-*L. racemosus* chromosome addition lines analyzed (Table 1).

### Confirmation of homoeologous groups of *L. racemosus* chromosomes in wheat background

To further assess the validity of our chromosome-specific SNP markers, we exploited correspondence of *L. racemosus* chromosome-specific markers with the homoeologous groups of CS chromosomes to determine the most probable homoeologous group (HG) of each *L. racemosus* chromosome in the chromosome addition lines (Table 2). The results revealed that the alien chromosomes spread between HG 2 and 7: Lr#A and L are in HG 2, Lr#H and N are in HG 3, while Lr#F, I, K and J are in HG 4, 5, 6 and 7, respectively.

**Table 2:** Determination of homoeologous groups of *L. racemosus* chromosomes in wheat genetic background using chromosome-specific DArT-seq SNP markers

Alien	Number of markers corresponding to each homoeologous group of bread wheat (Chinese Spring) chromosome																					Most probable HG of alien chromosome	
	1A	1B	1D	2A	2B	2D	3A	3B	3D	4A	4B	4D	5A	5B	5D	6A	6B	6D	7A	7B	7D	Previous reports	Current
Lr#A	3	1	1	**75**	**71**	**198**	3	0	4	0	2	2	1	0	5	1	3	5	2	3	1	2^a,b^	2
Lr#E	0	0	0	0	0	0	0	0	0	0	0	0	0	0	1	0	0	0	0	0	0	ND	ND
Lr#F	4	2	3	1	2	5	0	0	0	**77**	**79**	**141**	2	12	20	0	2	0	3	1	2	4^a,b^	4
Lr#H	1	2	7	0	2	2	**68**	**94**	**204**	2	2	4	3	1	2	0	6	2	0	4	4	3^a,b^	3
Lr#I	3	0	5	3	1	4	1	1	5	2	7	9	**74**	**162**	**193**	4	2	4	3	6	2	5^a,b^	5
Lr#J	1	0	3	4	2	5	2	2	4	12	2	4	5	7	2	0	3	5	**84**	**105**	**208**	(6,7)^a^, (3,7)^b^	7
Lr#K	4	2	4	1	3	7	1	2	2	0	2	6	2	2	6	**81**	**117**	**205**	5	8	9	6^a,b^	6
Lr#L	1	0	1	**135**	**158**	**293**	1	6	6	3	3	2	2	7	9	0	3	6	11	5	5	2^a,b^	2
Lr#N	4	6	3	0	1	4	**77**	**131**	**176**	3	0	0	1	3	3	0	2	1	2	4	5	2^a^, (3,7)^b^	3

*Lr Leymus racemosus*, *HG* Homoeologous group, *ND* Not determined; ^a^: [12]; ^b^: [9]

The bold numbers represent the number of markers indicating the homoeologous groups of *L. racemosus* chromosomes

### Detailed characterization of chromosomes I, J, N and their respective translocation arms

As shown in Table 3, we successfully allocated I-, J- and N-specific markers to their respective arms using their respective translocation lines.

**Table 3:** Determination of arm-specific markers of chromosomes Lr#I, Lr#J and Lr#N based on markers specific to their translocated arms

Genotype ID	Description	Chromosome constitution (2n)	Arm-specific markers		
			PCR	DArTseq	Total
I short	I short arm translocation	42	1	80	81
I long	I long arm translocation	42	0	404	404
J short	J short arm translocation	42	5	231	236
J long	J long arm translocation	42	0	2	2
N short	N short arm translocation	42	2	162	164
N long	N long arm translocation	42	3	255	258

**Fig. 2:**
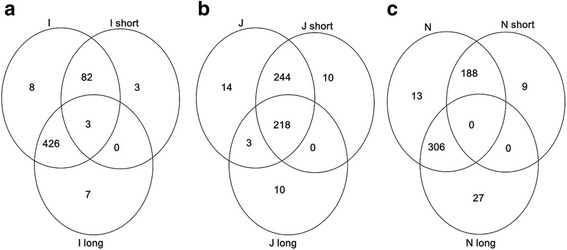
Venn diagrams showing homology and polymorphism between chromosomes I, J, N and their respective translocation lines. **a** homology and polymorphism between I and its translocated arms. **b** homology and polymorphism between J and its translocated arms. **c** homology and polymorphism between N and its translocated arms

A detailed analysis of the homology between each of the three chromosomes and their respective translocated arms gave a clearer picture of the structures of the translocation lines. For chromosome I, the markers were adequately allocated to the short (S) and long (L) arm translocations, revealing the proportions of chromosome I markers that differentiated each of the translocated arms and eight markers located on a segment of chromosome I that may have not been transmitted during the production of the translocation lines (Fig. 2a). However, we observed few markers specific to the translocation lines, which are absent in I-addition line. If no genotyping error is assumed, these markers would represent polymorphisms that may have arisen from the interactions between the translocated arms and CS genome.

Most J-chromosome markers were found to be present only in the JS translocation, about half of which were co-located on the JL translocation (Fig. 2b). This result obviously indicates that what we hitherto regarded as JL translocation is a segment of JS translocation. The 10 unique markers (Fig. 2b) each present in the two translocation lines may have resulted from changes in each genetic background or small chromosomal rearrangements during the various production processes. As observed in chromosome I, 14 markers identified a segment of chromosome J which may have not been transmitted to the translocation lines.

Chromosome N and its translocated arms presented a scenario similar to chromosome I. Both the NS and NL arm translocations of chromosome N showed well separated markers (Fig. 2c). However, 27 markers were found to be specific to NL and nine specific to NS translocation lines (Fig. 2c), indicating unique polymorphisms which may have been acquired from interactions between the background and the translocated arms as observed for chromosomes I and J. Also, the 13 markers located on the whole N-addition line, which are absent in the translocated arms, suggest that the NS and NL translocations lost the region of chromosome N identified by these markers (Fig. 2c).

### Analysis of recombination positions of N-recombination lines

**Fig. 3:**
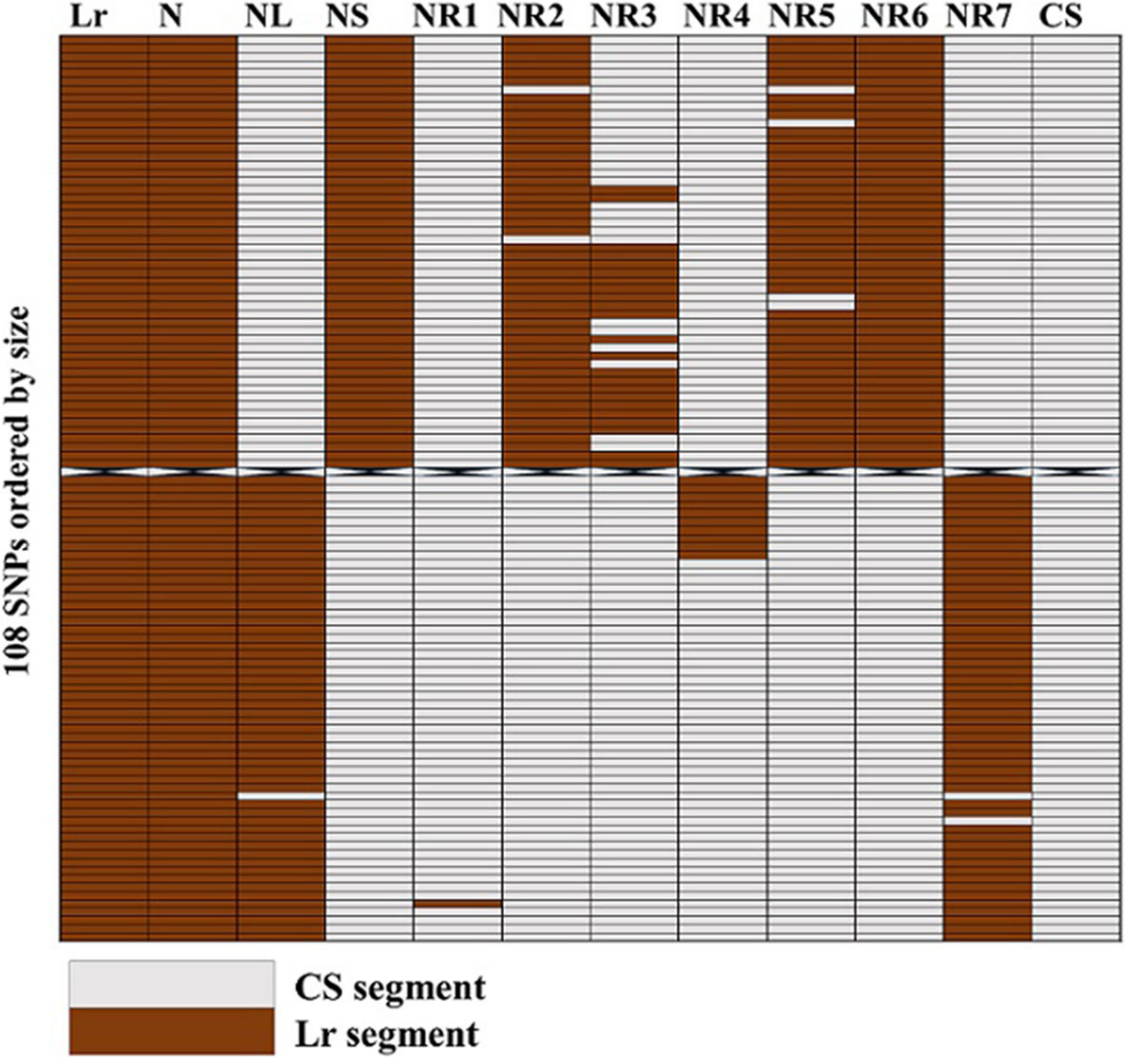
Graphical genotyping of N-recombination lines using 108 chromosome N-specific SNPs corresponding to wheat chromosome 3B. Lr: *Leymus racemosus*, N: N-addition line, NL: N long arm translocation line, NS: N short arm translocation line, NR1–NR7: N recombination lines 1–7, CS: bread wheat cv. Chinese Spring

The N-specific markers aided us to determine the size of the recombinant fragments and map their locations on Lr#N and the corresponding CS chromosomes, revealing the probable fraction of CS chromosome replaced in each recombination line (Table 4; Fig. 3). N recombinant fragments 2, 3, 5 and 6 were found to be located in the short arm, while the recombinant fragments 4 and 7 were found in the long arm of each of the lines. Although the two markers that specified recombinant fragment 1 were traced to NL, we are not certain about the arm location of this fragment; hence, we intend to clarify this in a future report. Recombination lines 6 and 7 were observed to have the largest fragments, with all the markers in the short arm translocation represented in recombination line 6, and all except two markers in long arm translocation represented in recombination line 7 (Table 4; Fig. 3). Other lines were found to have relatively small fragments which can best be described as different sizes of bins represented in recombination lines 6 or 7. With the two markers recorded for recombination line 1 (Table 4), it would appear as though there was no recombination event, although low recombination rates between wheat chromosomes and aliens is not unusual [24]. However, molecular cytogenetic characterization clearly differentiated the lines (Additional file 10), indicating the importance of integrated characterization of wheat-alien CILs.

**Table 4:** Determination of arm locations of N-recombinant fragments using arm-specific markers

Genotype	Arm location of amplified markers				Fragment location
	PCR		DArTseq		
	Short	Long	Short	Long	
N recombination No. 1	0	0	0	2	Not certain
N recombination No. 2	2	0	160	0	Short arm
N recombination No. 3	0	0	67	0	Short arm
N recombination No. 4	0	1	0	48	Long arm
N recombination No. 5	2	0	158	0	Short arm
N recombination No. 6	2	0	162	0	Short arm
N recombination No. 7	0	3	0	248	Long arm
N short arm translocation	2	–	162	–	–
N long arm translocation	–	3	–	255	–

### *Leymus racemosus* Chromosomes’ universal markers

**Fig. 4:**
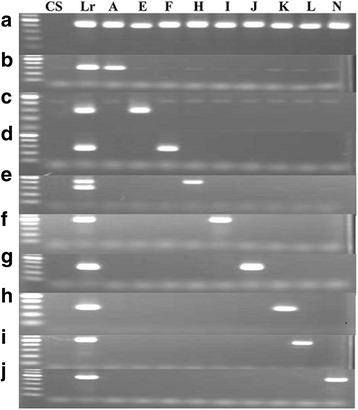
Representative gels of PCR amplification of wheat, *L. racemosus* and wheat-*L. racemosus* chromosome addition lines. **a** Amplification of nine *L. racemosus* chromosomes in wheat background by a universal marker. **b–j** Amplification of the nine chromosomes by their respective PCR-based chromosome-specific markers. CS: Chinese Spring; Lr: *Leymus racemosus*; A, E, F, H, I, J, L, N: Nine wheat-*Leymus racemosus* addition lines

Two of the polymorphic markers, *21_s46518* and *333_s46518* (developed from the same DNA sequence scaffold) identified all the *L. racemosus* chromosomes in wheat (Fig. 4a; Additional file 11). On sequencing PCR products generated with one of these markers, and conducting BLAST search with the official NCBI search tool (BLASTN, megablast), we observed that 26% and 16% of sequences of the PCR products of *L. racemosus* and wheat-Lr#N, respectively aligned to a section of CACTA-family transposon in *Lolium perenne* (perennial rye-grass), a highly researched commercial pasture crop of the grass family, Poaceae. Sequences of other CILs showed no significant alignment to the transposon sequence.

### Additional unique CILs-based SNPs

DArT-seq data further revealed additional 1468 unique SNPs in the nine wheat-*L. racemosus* addition lines, absent in the two parents. One hundred and ninety-seven of these SNPs are common to the lines, while 1271 are line-specific, with a range of 38–355 specific markers on each line (Table 5). Like the *L. racemosus* chromosome-specific markers, the line-specific markers also guided us in differentiating the nine addition lines. These additional SNPs account for polymorphisms acquired from the interactions between the added chromosomes and the background (CS genome), and their effects may be of agronomic significance.

**Table 5:** Additional 1468 unique genotype-based SNPs located on nine wheat-*Leymus racemosus* chromosome addition lines

Genotype ID	Alien chromosome ID	Common SNPs on each chromosome	Line-specific SNPs
TACBOW 0001	Lr#A	99	355
TACBOW 0003	Lr#E	79	38
TACBOW 0004	Lr#F	70	173
TACBOW 0005	Lr#H	86	109
TACBOW 0006	Lr#I	74	46
TACBOW 0007	Lr#J	68	130
TACBOW 0008	Lr#K	61	186
TACBOW 0009	Lr#L	92	124
TACBOW 0010	Lr#N	81	110
KT020–003 (CS)	–	0	0
TACBOW 0112 (Lr)	–	0	0
**Total**		**197**	**1271**

*TACBOW* Tottori Alien Chromosome Bank of Wheat (Japan), *CS* Chinese Spring, *Lr Leymus racemosus*;**197** represents the total number of SNPs common to the nine CILs, without repetition

### Analysis of transferability of *L. racemosus* markers to other Triticeae species

To assess the applicability of *L. racemosus* markers in studying the genomes of other related species, we analyzed the transferability of the markers using genomic DNA from 11 other Triticeae species, alongside with *L. racemosus*. The results of this analysis, utilizing 164 pre-screened *L. racemosus* PCR-based markers, showed that 75% of the markers were transferable, while the remaining 25% were *L. racemosus* genome-specific, particularly revealing higher amplification frequencies in three other important perennial Triticeae species (*L. mollis, Psathyrostachys huashanica* and *Elymus ciliaris*) in comparison to wheat and other species studied (5a-d; Table 6). More importantly, the amplified markers in each of these species were found to be reasonably polymorphic in wheat (Table 6), obviously indicating their suitability in genotyping wheat-alien CILs carrying chromosomes from these species.

**Table 6:** Transferability of 164 pre-screened *L. racemosus* markers to 11 other Triticeae species

Species	Amplified markers			Polymorphism to wheat (%)	Polymorphism based on marker source (%)	
	DNA	RNA	Total		DNA	RNA
*Leymus racemosus*	76	88	164 **(100)**	67	83	53
*L. mollis*	31	64	95 **(58)**	49	74	38
*Psathyrostachys huashanica*	23	55	78 **(48)**	44	70	33
*Elymus ciliaris*	17	49	66 **(40)**	32	42	29
*Hordeum vulgare*	5	31	36 **(22)**	22	0	16
*H. bulbosum*	8	31	39 **(24)**	23	25	23
*Dasypyrum villosum*	9	39	48 **(29)**	21	33	18
*Secale cereale*	9	38	47 **(29)**	15	11	16
*Triticum urartu*	9	36	45 **(27)**	7	0	8
*Aegilops speltoides*	8	39	47 **(29)**	6	13	5
*Ae. Tauschii*	10	39	49 **(30)**	0	0	0
*T. aestivum*	10	51	61 **(37)**	–	–	–
***L. racemosus-*** **specific markers**	33	8	41 **(25)**	–	–	–

Bold numbers in brackets represent percentages of amplified markers; *L. racemosus*-specific markers represent the proportion of the 164 markers not transferable to the other Triticeae species analyzed

**Fig. 5:**
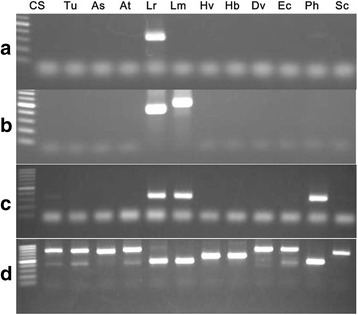
Representative gels of PCR amplification of 12 Triticeae species**. a** Amplification of *L. racemosus* by its genome-specific marker **b** Amplification of *Leymus* species by a *Leymus*-specific marker. **c** Specific amplification of *Leymus* species and *Psathyrostachys huashanica* (a species of *Leymus* N-genome progenitor genus). **d** Amplification of all the species by a conserved marker sequence, showing size polymorphism between the species

**Fig. 6:**
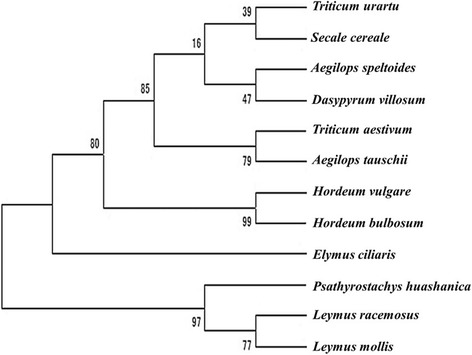
Phylogenetic tree constructed from the co-amplification of 123 PCR-based *Leymus racemosus* markers using UPGMA as clustering method

Interestingly, the two universal markers which identified all *L. racemosus* chromosomes in wheat genetic background were found to be *Leymus*-specific, as they amplified only the two *Leymus* species out of the 12 Triticeae species analyzed, revealing size polymorphism between the two *Leymus* genomes (Fig. 5b). These markers can, therefore, be applied to separate *Leymus* genomes from genomes of other species in the same tribe, and their (*Leymus*) chromosomes, if introgressed into wheat, can easily be sorted out in one PCR. We also observed informative co-amplification between the two *Leymus* species and *Psathyrostachys huashanica* (Fig. 5c), and a phylogenetic analysis using 123 markers co-amplified among the 12 Triticeae species (Fig. 6) revealed a close evolutionary relationship between the three species, which agrees with reports asserting that *Leymus* species are segmental polyploids with variant N-genomes from genus *Psathyrostachys* [13, 25]. However, one highly conserved marker sequence amplified all the species, revealing size polymorphism among them (Fig. 5d).

### Analysis of polymorphism based on DNA and RNA markers

In a bid to compare the performance of markers developed from DNA and RNA-seq, we analyzed polymorphism based on the two sources of markers. As expected, markers from genomic sequence were more polymorphic than those from RNA-seq, indicating that the polymorphisms between hexaploid wheat and other Triticeae species studied are more traceable to the variations in the repetitive sequences of the genomes (Table 6). However, the polymorphisms recorded from the RNA-seq/gene markers (Table 6), which account for variations in the genic regions, make the two approaches equally informative.

## Discussion

### Fast-tracking introgression breeding and wheat-alien characterization with appropriate molecular markers

Utilizing introgressive hybridization to combat the age-long wheat genetic erosion has since been identified and is currently inevitable, but the achievements are still not satisfactory mostly resulting from poor understanding of the genomics of important wild relatives of wheat [3, 26].Therefore, to create the necessary platform for successful breeding of hexaploid wheat through the intermediary of its tertiary gene pool, mobilization of research resources towards genome analysis and development of molecular markers from notable Triticeae perennial species must be intensified. The projected 60% increase in wheat demand in 2050 [27], which obviously cannot be met solely through the cultivation of high yielding elite wheat cultivars, most of which are poorly adapted to harsh growing conditions, further justifies our opinion.

From the stand point of our results (Tables 1–4; Fig. 3), it is evident that the availability of adequate molecular markers from the genomes of potential gene donors can accelerate introgression breeding, as they can be reliably deployed to genotype wheat-alien CILs and tackle linkage drag, where necessary. The massive chromosome-specific markers developed for each of the chromosome addition lines (except Lr#E) are, therefore, expected to aid breeders in conducting more stringent screening and selection in their efforts to develop cultivars with only necessary chromosome segments to satisfy specific breeding goals within a reasonable time frame. At the moment, we attribute the few chromosome-specific markers developed for Lr#E to high homology between wheat genome and the chromosome, having observed about 30% monomorphic markers between *L. racemosus* and *T. aestivum* genomes (Fig. 1a and c). However, there may be some form of genetic instability, cytochimerism for instance, which we intend to ascertain in the future. We are certain that alien chromosome loss is not the reason for the strange result, as we used the original stocks of all the addition lines in the TACBOW gene bank, which had been characterized by some of our co-authors [9, 28], to confirm our results.

The difference between the total number of markers developed and the number of markers that identified the aliens in wheat background (Table 1; Fig. 1b and d) account for the difference between the whole set of chromosomes in *L. racemosus* genome and the number of *L. racemosus* chromosomes we studied in the nine genotypes. Another factor likely to contribute to this difference is the possibility of losing some segments of the nine chromosomes during production of the lines.

Noteworthy is that the higher proportion of PCR markers that identified aliens in wheat as compared to SNP markers (Fig. 1b and d) was expected because SNP markers are sequence-based, which are theoretically more stringent than PCR-based markers. However, the proportions of both the PCR-based and SNP markers that amplified the nine chromosome addition lines are deemed reasonably high, given that *L. racemosus* has 14 pairs of chromosomes, 6 of which are obviously not represented in the nine genotypes analyzed. Also, the effectiveness of the chromosome-specific SNP markers in determining the homoeologous groups of *L. racemosus* chromosomes further validates the importance of inclusion of appropriate molecular markers in wheat-alien cultivar development and screening. The homoeologous groups of the chromosomes determined by our analysis are highly consistent with previous reports [9, 12]. Interestingly, we could clarify the homoeologous groups of chromosomes Lr#J (HG 7) and Lr#N (HG 3), which were previously not reported with certainty (Table 2). We presume that the chromosomes found to be in the same groups [Lr#A and Lr#L (HG 2); Lr#H and Lr#N (HG 3)] are homoeologous chromosomes from the two genomes that constitute *L. racemosus.* Our inability to determine the HG of Lr#E chromosome, as was the case with earlier reports cited here, is another pointer that the line may be genetically unstable.

In our opinion, this approach of utilizing chromosome-specific genome-based molecular markers to characterize introgression lines is faster than in situ hybridization procedures (FISH and GISH), which are traditionally employed for this purpose. Although in situ hybridization methods have proven to be reliable in characterizing CILs, they are lengthy, laborious and not without limitations. An example of such limitations was reported when two genomic in situ hybridization (GISH) procedures failed to reveal some distally located breakpoints in wheat-rye recombinant genotypes [29]. Beyond being easier, characterization of wheat-alien CILs by molecular markers, as proven by our results, brought to light detailed chromosome segments rearrangements, some of which can be likened to “zebra” chromosome [30, 31]. Additionally, the unique line-specific polymorphisms revealed by DArT-seq analysis, absent in either of the parents, would not be captured by in situ hybridization methods, as hybridization probes are usually designed to track alien segments, not polymorphisms which may arise from genome interactions. Nevertheless, we are not suggesting the replacement of hybridization procedures with molecular markers. Rather, our strong recommendation is the integration of efficient DNA markers with in situ hybridization strategies in wheat-alien breeding programs to accelerate the process and improve outcomes.

### Possibility of genome or alien modification in wheat-alien translocations

The interactions between alien chromosomes and carrier genomes need to be properly dissected. Analyses of the chromosome addition and translocation lines in our study indicated the possibility of genetic modification of either the introgressed chromosomes, background (wheat genome) or both. These modifications, capable of generating additional polymorphisms, as observed in our study (Table 5; Fig. 2), may result from small chromosomal rearrangements, activation of transposable elements or any other interactive genetic event between alien materials with the genome of wheat [31, 32]. Also, by graphically genotyping the recombination lines, we were able to uncover different patterns of recombination events in each line (Fig. 3). This observation indicates that the same chromosome (Lr#N) interacted with wheat genome in different ways to produce different genotypes, which are likely to result in diversity in agronomic traits. Of more importance is the potential effect of these interactions on the overall performance of the genotypes [33], necessitating detailed studies to clarify the underlying mechanism of such genetic events and their agronomic implications. Such studies would be greatly enhanced by the availability of adequate molecular markers to track aliens and unique polymorphisms which may result from genome interactions.

### Association of *L. racemosus* chromosomes’ universal markers with CACTA-family transposons

The universal markers we developed are particularly valuable since they can be applied to easily track the transmission of alien chromosomes over generations, given the possibility of alien chromosome elimination in the course of cultivar multiplication and maintenance [34–36]. Following the alignment of the sequence of one of these markers to CACTA-family transposon in *L. perenne*, we speculate that this marker sequence is part of a possible CACTA-family transposon in *Leymus*. CACTA-family transposons, one of the most abundant superfamilies of class II transposons exclusively found in plants, have been reported to play significant roles in genome variation in Triticeae and other plants [37–41]. Although the specific role of this sequence in *Leymus* species is unknown at the moment, it is likely to have amplified after differentiation of the ancestral species of *Leymus*.

### *Leymus* chromosome N-specific markers and biological nitrification inhibition (BNI) activity

Biological nitrification inhibition (BNI) activity in *L. racemosus,* a highly desirable trait with agronomic and environmental consequences, had previously been reported to be chiefly controlled by chromosome N [42]. The N-specific markers are, therefore, particularly of high value, as they can easily be applied to identify genotypes with BNI activity, avoiding the cumbersome and expensive process of root exudates analysis [43], requiring expertise which an ordinary plant breeder may not possess. Interestingly, only DNA sequences of the PCR products of *L. racemosus* and wheat-Lr#N generated with one of our universal markers aligned to the CACTA-family transposon in *L. perenne,* one of the forage grasses reported to have endogenous BNI activity [42, 43]. However, whether BNI activity is linked with actions of mobile genetic elements, transposons in this case, cannot be ascertained at the moment.

### Transferability of markers between *L. racemosus* and other Triticeae perennials

Sequencing of all the potential gene sources for wheat breeding in the near future is not expected. Hence, transferability of markers between useful species of this gene pool, as a compensational approach of analysis, is highly desired [44–47]. Our analysis has proven clearly that markers from *L. racemosus* can be successfully transferred to *L. mollis, P. huashanica* and *E. ciliaris*, three other important species in the tribe Triticeae (Table 6). Also, the transferred markers were found to be reasonably polymorphic in wheat (Table 6), suggesting their suitability for characterizing wheat genotypes with alien chromosomes from the three genomes. The genera of these species, because of their recognition as profitable forage grasses and gene mines for hexaploid breeding, have received fair research attention [13, 14, 23, 25, 48–50]. However, their genomes have not yet been sequenced, leaving breeders with the option of transferring markers from evolutionary closely related species to analyze their genomes and wheat genotypes carrying their chromosomes.

## Conclusion

The molecular markers developed in this study are expected to play valuable roles in hexaploid wheat breeding, particularly in the process of developing and characterizing wheat-alien CILs. Our success in applying them to unequivocally genotype 22 wheat-*L .racemosus* CILs validates their usefulness. Specifically, the universal and N-specific markers are of great breeding importance. While the universal markers can readily be applied to monitor and confirm alien presence and transmission, N-specific markers can find application in mapping of nucleotide sequences associated with biological nitrification inhibition (BNI) activity. The additional SNPs found on the nine chromosome addition lines would be especially useful in identifying and analyzing unique polymorphisms which may result from alien interaction with background, while the *L. racemosus* markers not mapped on any of the nine chromosomes reported here would aid production of other wheat-*L. racemosus* CILs carrying other chromosomes of *L. racemosus***.** Also, the remarkable transferability of the PCR-based markers to three other notable perennial Triticeae species is an added advantage, as they can be deployed to characterize wheat-alien CILs bearing chromosomes from these genomes. Since this is the first report on the development of molecular markers from this genome, coupled with the efficiency of the markers as proven in our results, we recommend wide application of these markers in bread wheat breeding programs. Integrating the markers with in situ hybridization strategies would undoubtedly shorten the duration of cultivar development and produce more reliable results.

## Methods

### Plant materials

We analyzed 22 wheat-*L. racemosus* CILs (Table 7), three cultivated and nine wild Triticeae species (Table 8). The nine chromosome addition lines and 12 Triticeae species were obtained from the gene bank of Tottori Alien Chromosome Bank of Wheat (TACBOW), a subsidiary of the National BioResource Project (NBRP)-KOMUGI, Japan, while the six translocation and seven recombination lines were personally provided by Dr. M. Kishii (co-author) of the International Maize and Wheat Improvement Center (CIMMYT). Apart from the two *Leymus* species and *Elymus ciliaris*, which are maintained permanently as live plants in Arid Land Research Center (ALRC), Tottori University, seeds of all other experimental plants were sown in trays filled with commercial soil (Takii and CAINZ, Japan), and the germinated plants were maintained until leaf samples were ready for DNA extraction. About two weeks after sowing, leaf samples were collected from each plant, immediately frozen in liquid nitrogen and stored at –80 °C until needed for DNA extraction. Cetyl trimethylammonium bromide (CTAB) miniprep extraction protocol, with some modifications, was followed to extract and purify genomic DNA from all samples, while quantification and quality check were done with NanoDrop2000C Spectrophotometer (ThermoScientific, USA).

**Table 7:** Description and references of wheat-*L. racemosus* chromosome introgression lines (CILs)

Genotype ID	Description	Alien chromosome ID	Chromosome constitution (2n)	Reference
TACBOW 0001	Disomic addition	Lr#A	21″ + 1″[A]	[9]
TACBOW 0003	Disomic addition	Lr#E	21″ + 1″[E]	[9]
TACBOW 0004	Disomic addition	Lr#F	21″ + 1″[F]	[9]
TACBOW 0005	Disomic addition	Lr#H	21″ + 1″[H]	[9]
TACBOW 0006	Disomic addition	Lr#I	21″ + 1″[I]	[9]
TACBOW 0007	Disomic addition	Lr#J	21″ + 1″[J]	[9]
TACBOW 0008	Disomic addition	Lr#K	21″ + 1″[K]	[9]
TACBOW 0009	Disomic addition	Lr#L	21″ + 1″[L]	[9]
TACBOW 0010	Disomic addition	Lr#N	21″ + 1″[N]	[9]
I short	I short arm translocation	Lr#IS	42	[51]
I long	I long arm translocation	Lr#IL	42	[51]
J short	J short arm translocation	Lr#JS	42	[51]
J long	J long arm translocation	Lr#JL	42	[51]
N short	N short arm translocation	Lr#NS	42	[51]
N long	N long arm translocation	Lr#NL	42	[51]
N recomb #1	N recombination No. 1	Lr#NR1	42	Current article
N recomb #2	N recombination No. 2	Lr#NR2	42	Current article
N recomb #3	N recombination No. 3	Lr#NR3	42	Current article
N recomb #4	N recombination No. 4	Lr#NR4	42	Current article
N recomb #5	N recombination No. 5	Lr#NR5	42	Current article
N recomb #6	N recombination No. 6	Lr#NR6	42	Current article
N recomb #7	N recombination No. 7	Lr#NR7	42	Current article

*TACBOW* Tottori Alien Chromosome Bank of Wheat (Japan), *Lr Leymus racemosus*, *A, E, F, H, I, J, K, L, N* Arbitrary numbering of *L. racemosus* chromosomes, *IS* I-short arm, *IL* I-long arm, *JS* J-short arm, *JL* J-short arm, *NS* N-short arm, *NL* N-long arm, *NR* N-recombination

**Table 8:** Description and sources of 12 Triticeae species

Species ID	Description	2n	Ploidy	Reference
KT020–003	*Triticum aestivum* cv*. ‘*Chinese Spring’	42	6×	[18]
TACBOW0071	*Secale cereale* strain IR10	14	2×	–
TACBOW0112	*Leymus racemosus*	28	4×	[28]
TACBOW0113	*L. mollis*	28	4×	[28]
TACBOW0116	*Hordeum vulgare* cv.’Betzes’	14	2×	[18]
TACBOW0117	*H. bulbosum*	14	2×	[18]
TACBOW0119	*Dasypyrum villosum*	14	2×	[18]
TACBOW0121	*Psathyrostachys huashanica*	14	2×	[10]
TACBOW0122	*Elymus ciliaris*	28	4×	[18]
KU-199-1	*T. urartu*	14	2×	–
KU-2-1	*Aegilops speltoides*	14	2×	–
KU-20-2	*Ae. Tauschii*	14	2×	–

*TACBOW* Tottori Alien Chromosome Bank of Wheat (Japan). All the strains and their detailed description and origin are available in the gene bank of National BioResource Project (NBRP-KOMUGI) (https://shigen.nig.ac.jp/wheat/komugi/)

### Production of wheat-*L. racemosus* chromosome introgression lines

Details of the production procedures and identification of *L. racemosus* chromosomes in the chromosome addition and translocation lines were reported in previous studies [9, 28, 51]. We, therefore, report here the additional steps taken to develop the N-recombination lines, which we characterized alongside with the addition and translocation lines in this study. Basically, our strategy was modelled after the methodology described and adopted in the production of wheat-rye recombination lines [24]. To produce the first N-recombination line, N recomb #1, we crossed a monosomic Chinese Spring (CS) wheat line (2n = 42 – 3B’) to a disomic N-addition line (2n = 42 + N″) and selected N monosomic substitution plants (2n = 42 + N′ – 3B’) in the first filial generation (F_1)_. In the second filial generation (F_2)_, a naturally occurring recombinant was recognized by FISH/GISH analysis and homozygote recombinants were selected and named N-recomb #1. The production of N-recomb #2 to #7, except #4, was initiated by the hybridization of N-short arm translocation with a CS *ph 1* mutant (CS-*ph 1*) to enable homoeologous pairing and recombination. The F_1_ plants were then self-pollinated and five sets of recombinant homozygotes, with different recombination rates, were selected. For N-recomb #4, we crossed another bread wheat cultivar, WEBILL1 (a dominant bread wheat line at CIMMYT in the 2000’s), with a disomic N-addition and selected monosomic addition plants at the F_1_, which were then backcrossed to WEBILL1 to generate BC_1_F_1_ monosomic addition plants. The BC_1_F_1_ monosomic plants were then hybridized with CS-*ph 1* mutant and N-recomb #4 homozygote plants were selected at the F_2_, following the steps described for the last five genotypes.

### Growth conditions for *Leymus racemosus* seedlings used for sequencing

Seedlings of *Leymus racemosus* were raised in a hydroponic culture (2 mM KNO_3_, 0.28 mMKH_2_PO_4_, 0.18 mM K_2_SO_4_, 0.07 mM CaCl_2_・2H_2_O, 0.15 mM MgSO_4_・7H_2_O, 75 nM H_3_BO_3_, 12.5 nM MnSO_4_・6H_2_O, 25 nM Na_2_MoO_4_・2H_2_O, 10 nM ZnSO_4_・7H_2_O, 0.5 mM Fe-EDTA, 5 nM CuSO_4_・5H_2_O), with a day/night temperature regime of 25/15 °C (14 h light/10 h dark photoperiod) in Arid Land Research Center growth chamber for two months. Hydroponic solution was changed every week. To obtain young root tissues*,* old-lignified roots were partially cut and plants were grown in the hydroponic culture for two weeks. Young roots were then separately subjected to salinity stress and ammonium treatment for 12 h, to ensure gene expression for salinity tolerance and BNI activity, which are reported traits of *L. racemosus* [17, 42]. Salinity stress was imposed by addition of 400 mM NaCl to the hydroponic medium, while ammonium treatment was achieved by replacement of 2 mM KNO_3_ with 2 mM (NH_4_)_2_SO_4_. Control plants were maintained in unaltered hydroponic medium. Root tissues were harvested, frozen in liquid nitrogen and stored at –80°Cuntil needed for DNA and RNA extraction.

### RNA extraction and library preparation

Control-, salt- and ammonium-treated root tissues of *L. racemosus* were used for RNA-sequencing. Total RNA was extracted using RNeasy mini kit with the inclusion of an on-column DNase digestion kit (Qiagen). Using the isolated RNA, mRNA-seq libraries were constructed for the three conditions using TruSeq RNA Sample Preparation. To generate 150-bp pair-end reads, the libraries were sequenced by HiSeq2500 according to the standard protocol.

### Assembly of RNA-sequencing reads

A total of 174-GB reads were determined by mRNA-sequencing (Additional file 12). Approximately 5% of reads with low-quality scores or adapters were partially trimmed by Trimmomatic software (version 0.32) [52]. To remove non-mRNA sequences, we collected known rRNA and tRNA sequences [53, 54], and after removing the reads mapped to rRNA and tRNA sequences by Bowtie2 software (version 2.2.3) [55], the remaining reads were used for construction of assembled contigs in either control, high ammonium or salinity stress condition. First, assembled contigs were generated by three softwares: Velvet ver. 1.2.10-Oases ver. 0.2.8., SOAPdenovo-Trans Ver. 1.0.3 and Trinity ver. 2.1.1 [56] in sequencing reads derived from control condition. Contigs assembled by Velvet-Oases used various kmer-sizes (39, 49, 59, 69, 79, 89 and 99)(Velvet version 1.2.10, Oases version 0.2.8), and all the contigs assembled by various kmer-sizes were merged into a contig. Contigs assembled by either SOAPdenovo-Trans or Trinity used optimized K-mers. There are three assembled contigs in Velvet-Oases, SOAPdenovo-Trans and Trinity. To identify the best contig among three contigs, we examined the conserved regions of *L. racemosus* against *T. aestivum* cDNA sequences (version 30, Ensembl) by BLASTN software [57]. The proportions of conserved sequences were 44%, 17% and 4% in Velvet-Oases, SOAPdenovo-Trans and Trinity contigs, respectively. Here, Velvet-Oases generated the best contigs with respect to similarity in closely related species. In the same procedure, we generated 634,480, 460,748 and 434,862 contigs with more than 500 bp in normal, high ammonium and salinity stress condition, respectively.

### Primer design from RNA-seq

We designed primers in the homologous regions between *T. aestivum* and *L. racemosus* and *L. racemosus* specific region*,* resulting in two categories of primers. First, both forward and reverse primers are in the homologous regions but the length of amplified DNA fragment is expected to be different between *T. aestivum* and *L. racemosus* by 20–1000 bp*.* Second, either forward or reverse primer is in the homologous region, while the other primer is in *L. racemosus* specific sequence, and the amplified DNA fragment is expected to range from 100 to 1000 bp. All the primers were designed by Primer3 software [58]. We designed 9256 and 7637 pairs of primers in the contigs with more than 500 bp in normal condition in the former and latter strategies, respectively. To design primers in high salinity and ammonium contigs, we first removed high salinity contigs which are similar to normal condition contigs and then removed high ammonium contigs which are similar to either normal or high salinity contigs. From the remaining high salinity contigs, using the two strategies in the order explained above, we designed 4930 and 3339 pairs of primers, respectively, and from the filtered high ammonium contigs, we designed 5461 and 4312 pairs of primers, respectively. Thus, we identified a total of 34,935 pairs of primers to identify the difference between *T. aestivum* and *L. racemosus.*

### Genome sequencing and assembly

Genomic DNA was extracted from root tissues of 2-week-old *L. racemosus* plants (control treatment) using MagExtractor™ -*Plant Genome*- (TOYOBO). The isolated DNA was submitted to generate sequencing library for Illumina MiSeq analysis, and the library construction and sequencing process were achieved by a purchasable service from Macrogen, Japan. A total of ~ 35 M paired-end reads (2 × 151-nt) was obtained from the analysis. Subsequent quality trimming (Q > 30) and artificial sequence elimination steps were achieved manually. The cleaned reads were subjected to build *L. racemosus* genome contigs utilizing Platanus software (v1.2.4) [59]. Assemblies with variable k-mers (27, 29, 31, 33, 35, and 37) were conducted in parallel, and the resultants were merged into *L. racemosus* genome scaffolds of unique and significantly long (> 1000-nt) length. The raw sequence data was deposited in NCBI/EBI/DDBJ short read archive under a specific accession number (SRR5796629).

### Primer design from genomic sequence

Wheat mRNA-seq data from a previous study [60] was mapped to the *L. racemosus* genome scaffolds with the aid of TopHat (ver. 2.0.8) [61] with the following options: “--read-realign-edit-dist 0 –b2-fast –meta-std-dev 200 –a 6 -i 8 -I 10000 --max-segment-intron 100 --min-segment-intron 3”. Ten scaffolds were retained based on their length (> 2000-nt) and read mapping (no specific mRNA-seq read mapping). Scaffolds polymorphisms against wheat reference genome (v1.1) [62] were evaluated by Blastn, and primers sensitive to relatively large (> 3-nt) gaps were designed by Primer3.

### Development of markers and genotyping of wheat-*L. racemosus* chromosome introgression lines by PCR

Utilizing genomic DNA samples from bread wheat and *L. racemosus,* we screened 294 randomly selected primer sets – 150 from DNA sequence and 144 from RNA-seq. Each 20 *µ*L reaction volume contained 10 *µ*L KAPA Taq Extra HotStart ReadyMix with dye (KapaBiosystems), 1 *µ*L (10 *µ*M) each of forward and reverse primers, 2 *µ*L (50 ng) DNA template and 6 *µ*L PCR grade water. With BIORAD T100 Thermal Cycler, the samples, in a 96-well plate, were subjected to touchdown PCR: 95 °C initialization for 3 min, 5 cycles of 95 °C denaturation for 30 s, 65 °C to 61 °C (-1 °C/cycle) annealing for 30 s and 72 °C extension for 30 s; 30 cycles of 95 °C denaturation (30 s), 60 °C annealing (30 s) and 72 °C extension (30 s) and final extension at 72 °C for 10 s. Because the average melting temperature (Tm) of primers designed from DNA sequence was about 8 °C higher than the average Tm of primers designed from RNA-seq, we substituted the annealing range of 65-60 °C in the cycling program with 57-52 °C for the RNA-seq primers. All PCR products were electrophoresed for 30 min on 1.5% agarose S gel in Tris-acetate-EDTA (1X TAE) buffer, stained in ethidium bromide solution for 10 min and photographed with AE-6932GXCF transilluminator.

From the pre-screened primers, polymorphic markers were selected and applied to genotype nine wheat-*L. racemosus* chromosome addition lines and markers specific to *L. racemosus* chromosomes I, J and N were deployed to characterize two each of I-, J- and N-translocation and seven N-recombination lines, respectively. To assess the transferability of *L. racemosus* markers to other Triticeae species, we used 164 markers amplified in *L. racemosus* to genotype 12 species in the tribe (Table 8), including *L. racemosus* as a positive control, aiming at analyzing polymorphism between bread wheat genome and genomes of other species studied.

### Sequencing and analysis of some PCR products

We applied Sanger sequencing to determine the nucleotide sequence of PCR products generated by one of our markers which amplified all the *L. racemosus* chromosomes added to wheat. All the PCR products were purified with AxyPrep PCR cleanup kit, according to the PCR cleanup spin protocol (AXYGEN Biosciences). The purified products were premixed in accordance with Macrogen’s recommendation (Macrogen, Japan) and same delivered to the company for sequencing. Each genotype sequence was searched against nucleotide sequences in NCBI and Ensembl Plants databases using BLASTN (megablast). Also, the DNA scaffold from which the marker was developed was searched in like manner. To check for polymorphism between the chromosomes, we aligned all the sequences using JustBio multiple alignment tool.

### Development of markers and genotyping of wheat-*L. racemosus* chromosome introgression lines by DArT-seq

To complement the PCR-based markers and widen the scope of application of our makers, especially chromosome-specific markers, we applied DArT-seq to genotype *L. racemosus* and the 22 CILs alongside bread wheat to assist in data analysis and interpretation. DArT-seq platform used HiSeq2500 to sequence the samples and generated 44,277 markers. This approach enabled us to develop massive chromosome-specific markers for the nine *L. racemosus* chromosomes analyzed.

### Analysis of data

#### PCR data

PCR results were scored in a binary fashion, “0” and “1” for absence and presence of band, respectively, while size polymorphic bands (very few) were differentiated using 1 to designate band size in wheat and 2 for band size in an introgression line or another Triticeae species, depending on the case. The scores were analyzed using simple proportion to determine the percentage of screened primers amplified in *L. racemosus* genome as well as the proportion of the amplified markers polymorphic in wheat. Also, the frequency of amplification of alien chromosomes in the CILs was computed, with a view to making clear the proportion of the developed markers located on the alien chromosomes. Markers specifically amplified by each of the nine chromosome addition lines were designated chromosome-specific and I-, J- and N-specific markers specifically located on any of the translocation lines were accordingly named arm-specific. Also, arm-specific markers of chromosome N specifically amplified by the seven N-recombination lines were applied to determine the arm location of each recombinant fragment. Data from the screening of the 12 Triticeae species were handled in a similar manner, but with more emphasis in identifying and computing polymorphism in wheat in each case. This gave a basis to decide the suitability of *L. racemosus* for genotyping of wheat lines carrying chromosomes from these species. In addition, we used PCR-based markers amplified in each species to compare frequency of polymorphism between DNA and RNA sequence information to assess the suitability of the two approaches.

#### DArT-seq data

DArT-seq markers in the SNP 1-Row Mapping Format, which we used for our analysis, were scored “0”, “1” and “2”, representing reference (Wheat_ChineseSpring04) allele only, SNP allele only and both reference and SNP alleles, respectively. This simply means that any genomic representation scored “0” for a marker lacks the alien chromosome identified by the corresponding SNP allele, while those scored “1” or “2” have the SNP or both the SNP and reference alleles, respectively. We set the call rate at greater than or equal to 85% to aid us filter reliable markers from the stream of the entire data obtained from DArT-seq platform. Markers with no clear information regarding their physical positions on corresponding wheat chromosomes, no polymorphism in wheat genome and those without SNP alleles in *L. racemosus* were filtered against. In order to obtain the fraction of the polymorphic markers that provided useful genotypic information on the CILs, we sieved all the markers located on genomic representations of the lines, carefully separating chromosome-specific markers from co-amplified. We also analyzed homology between I, J, N and their respective translocation lines, aiming at identifying markers specific to each of the arms as done with the PCR markers and determining the locations of the N-recombinant fragments to allow comparison with the PCR-based result. With the corresponding physical positions of the N-specific markers revealed by DArT-seq data, we graphically genotyped the N-recombination lines. In the final analysis, correspondence of all the chromosome-specific markers with the homoeologous groups of CS chromosomes was utilized to determine the most probable homoelogous groups of *L. racemosus* chromosomes in the CILs.

## Additional files

Additional file 1: **Table S1.** Detailed information of Lr#A-specific markers. (XLSX 44 kb)

Additional file 2: **Table S2.** Detailed information of Lr#E-specific markers. (XLSX 10 kb)

Additional file 3: **Table S3.** Detailed information of Lr#F-specific markers. (XLSX 39 kb)

Additional file 4: **Table S4.** Detailed information of Lr#H-specific markers. (XLSX 40 kb)

Additional file 5: **Table S5.** Detailed information of Lr#I-specific markers. (XLSX 49 kb)

Additional file 6: **Table S6.** Detailed information of Lr#J-specific markers. (XLSX 44 kb)

Additional file 7: **Table S7.** Detailed information of Lr#K-specific markers. (XLSX 46 kb)

Additional file 8: **Table S8.** Detailed information of Lr#L-specific markers. (XLSX 52 kb)

Additional file 9: **Table S9.** Detailed information of Lr#N-specific markers. (XLSX 44 kb)

Additional file 10: **Fig. S1.** GISH photos showing molecular cytogenetic identification of alien segments in N-recombination lines. (PPTX 7852 kb)

Additional file 11: **Table S10.** Sequences of *Leymus racemosus* chromosomes’ universal markers. (DOCX 14 kb)

Additional file 12: **Table S11.** Raw mRNA sequence reads of *L. racemosus*. (DOCX 15 kb)

## Abbreviations

CTAB: Cetyl trimethylammonium bromide
DArT-seq: Diversity arrays technology-sequence based
DNA: Deoxyribonucleic acid
EDTA: Ethylene diaminetetraacetic acid
PCR: Polymerase chain reaction
RNA: Ribonucleic acid
TACBOW: Tottori alien chromosome bank of wheat
