# Supplementary material for: Efficient anchoring of alien chromosome segments introgressed into bread wheat by new Leymus racemosus genome-based markers
Source: BMC Genet. 2018 Mar 27;19:18. doi: 10.1186/s12863-018-0603-1 (PMC5872505; doi:10.1186/s12863-018-0603-1)
Supplement: Supplementary file 10 — Fig. S1. GISH photos showing molecular cytogenetic identification of alien segments in N-recombination lines. (PPTX 7852 kb) [file 12863_2018_603_MOESM10_ESM.pptx]

## Slide 1
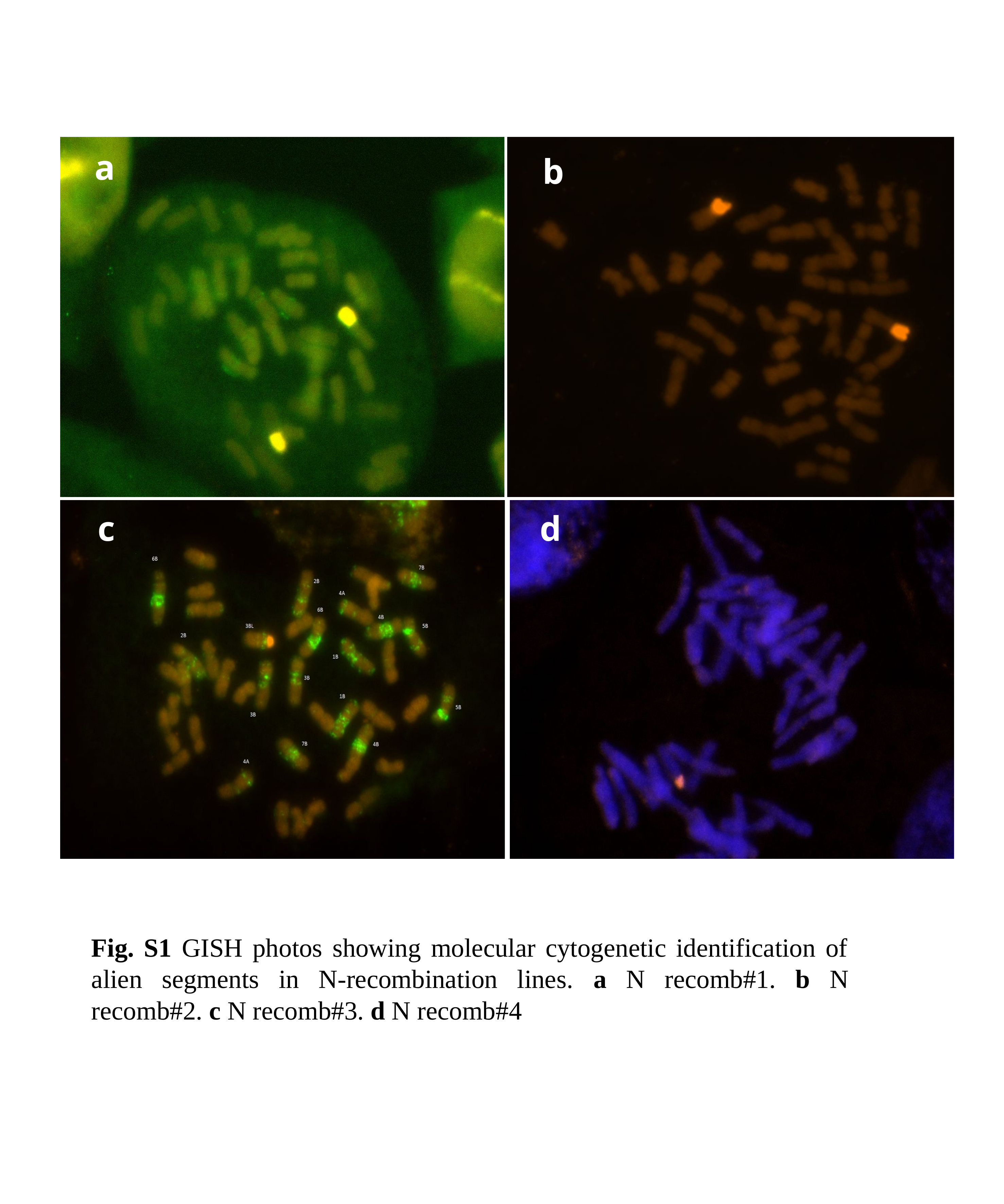

a
b
d
c
Fig. S1 GISH photos showing molecular cytogenetic identification of alien segments in N-recombination lines. a N recomb#1. b N recomb#2. c N recomb#3. d N recomb#4
