# Supplementary material for: Efficient anchoring of alien chromosome segments introgressed into bread wheat by new Leymus racemosus genome-based markers
Source: BMC Genet. 2018 Mar 27;19:18. doi: 10.1186/s12863-018-0603-1 (PMC5872505; doi:10.1186/s12863-018-0603-1)
Supplement: Supplementary file 11 — Table S10. Sequences of Leymus racemosus chromosomes’ universal markers. (DOCX 14 kb) [file 12863_2018_603_MOESM11_ESM.docx]

**Table S10** Sequences of *Leymus* *racemosus* chromosomes’ universal markers

| **Marker ID** | **Sequence** | **Tm** | **Product size** |
| --- | --- | --- | --- |
| 21_s46518f | AATGAATCAACATTTCGGCGATAACGG | 65.3 |  |
| 21_s46518r | CAGTGGGCATTTTACTTGGCAATTGAT | 65.3 | 284 |
| 333_s46518f | GTCATTTCAGTGGTAATGGGTGGGTAA | 66.1 |  |
| 333_s46518r | CAGTGGGCATTTTACTTGGCAATTGAT | 65.3 | 418 |
