# Supplementary material for: Efficient anchoring of alien chromosome segments introgressed into bread wheat by new Leymus racemosus genome-based markers
Source: BMC Genet. 2018 Mar 27;19:18. doi: 10.1186/s12863-018-0603-1 (PMC5872505; doi:10.1186/s12863-018-0603-1)
Supplement: Supplementary file 12 — Table S11. Raw mRNA sequence reads of L. racemosus. (DOCX 15 kb) [file 12863_2018_603_MOESM12_ESM.docx]

**Table S11** Raw mRNA sequence reads of *L. racemosus*

| **Treatment** | **Number of raw reads** | **Total length of raw reads** | **Number of trimmed reads** | **Total length of trimmed reads** |
| --- | --- | --- | --- | --- |
| Normal | 573305334 | 57330533400 | 546805162 | 54680516200 |
| Salinity | 640232346 | 64023234600 | 608428790 | 60842879000 |
| High ammonium | 529193164 | 52919316400 | 504436608 | 50443660800 |
